# Immune- and metabolism-related gene signature analysis uncovers the prognostic and immune microenvironments of hepatocellular carcinoma

**DOI:** 10.1007/s00432-024-05849-5

**Published:** 2024-06-19

**Authors:** Yange Gu, Ensi Ma, Shengran Jiang, Zhenyu Shan, Guixi Xia, Rui Ma, Jiaqi Fu, Zhengxin Wang

**Affiliations:** 1 Liver Transplantation Center, General Surgery, Huashan Hospital, Fudan University, Shanghai, China; 2 Institute of Organ Transplantation, Fudan University, Shanghai, China; 3 Bengbu Medical University, Bengbu, China

**Keywords:** Hepatocellular carcinoma (HCC), Immunity, Metabolic reprogramming, Signature, Tumour microenvironment (TME)

## Abstract

**Background:**

Metabolic reprogramming is an emerging hallmark that influences the tumour microenvironment (TME) by regulating the behavior of cancer cells and immune cells. The relationship between metabolism and immunity remains elusive. The purpose of this study was to explore the predictive value of immune- and metabolism-related genes in hepatocellular carcinoma (HCC) and their intricate interplay with TME.

**Methods:**

We established the immune- and metabolism-related signature (IMRPS) based on the LIHC cohort from The Cancer Genome Atlas (TCGA) dataset. Kaplan–Meier analysis, receiver operating characteristic (ROC) curve analysis and Cox regression analysis confirmed the prognostic value of IMRPS. We investigated differences in immune cell infiltration, clinical features, and therapeutic response between risk groups. The quantitative real-time PCR (qPCR) was used to confirm the expression of signature genes. Immunohistochemical staining was performed to evaluate immune infiltration features in HCC tissue samples. We conducted cell experiments including gene knockout, cell counting kit-8 (CCK-8), and flow cytometry to explore the role of the IMRPS key gene UCK2 in HCC. RNA-seq was used to further investigate the potential underlying mechanism involved.

**Results:**

The IMRPS, composed of four genes, SMS, UCK2, PFKFB4 and MAPT, exhibited significant correlations with survival, immune cell infiltration, clinical features, immune checkpoints and therapeutic response. The IMRPS was shown to be an excellent predictor of HCC prognosis. It could stratify patients appropriately and characterize the TME accurately. The high-risk HCC group exhibited an immunosuppressive microenvironment with abundant M_2_-like macrophage infiltration, which was confirmed by the immunohistochemistry results. The results of qPCR revealed that the expression of signature genes in 20 HCC tissues was significantly greater than that in adjacent normal tissues. After the key gene UCK2 was knocked out, the proliferation of the Huh7 cell line was significantly inhibited, and monocyte-derived macrophages polarized towards an M1-like phenotype in the coculture system. RNA-seq and GSEA suggested that the phenotypes were closely related to the negative regulation of growth and regulation of macrophage chemotaxis.

**Conclusions:**

This study established a new IMRS for the accurate prediction of patient prognosis and the TME, which is also helpful for identifying new targets for the treatment of HCC.

**Supplementary Information:**

The online version contains supplementary material available at 10.1007/s00432-024-05849-5.

## Introduction

Currently, liver cancer is the second leading cause of cancer-related deaths worldwide (Forner et al. 2018; GBD 2013 Mortality and Causes of Death Collaborators 2015). Hepatocellular carcinoma (HCC) accounts for 90% of liver cancer cases (Torre et al. 2015; European Association For The Study Of The Liver; European Organisation For Research And Treatment Of Cancer 2012), and an increase in major risk factors has led to a continuous increase in its incidence (El-Serag 2011).When curative therapy is limited, the administration of drugs for the treatment of unresectable advanced HCC does not significantly improve overall survival (OS) rates. Even first-line drugs such as lenvatinib, which targets cell growth and angiogenesis, can extend the survival period by only a few months because the tumours eventually progress (Kudo et al. 2018). Recently, immunotherapy has become the first-line treatment for advanced HCC, significantly improving the OS of unresectable HCC patients who had not received systemic therapy (Kelley et al. 2021; Llovet et al. 2022; Sangro et al. 2021). However, despite these advances in immunotherapy, HCC remains a highly lethal disease due to the acquisition of tumour resistance and its continuously increasing incidence and mortality rates (Llovet et al. 2022; Yang et al. 2023).

Specific enrichment of T-cell exhaustion, M2 macrophages and immunosuppressive regulatory T-cell signatures was found in samples from patients with metabolic dysregulation, which suggests that a suppressive tumour microenvironment (TME) exists (Beckermann et al. 2017; Ramapriyan et al. 2019; Sun et al. 2022; Zhang et al. 2022). Understanding the intricate interplay between metabolism and immunity, as well as its impact on cancer development and progression, remains a major challenge (Xia et al. 2021). It is essential to evaluate the prognosis, TME and drug sensitivity of HCC patients by utilizing the combined features of immune and metabolic data.

In this study, we simultaneously considered the impact of immune and metabolic factors on HCC and constructed an immune- and metabolism-related model by analysing genes related to immunity and metabolism. Cell experiments, including gene knockout, CCK-8 and flow cytometry assays, were also conducted to explore the role of the key gene UCK2 in HCC. QPCR assays of clinical samples were performed to verify the expression of the four genes in the model. Overall, our study may be helpful for predicting HCC prognosis, immune status and treatment response and was validated for guiding clinical therapy and predicting patient prognosis.

## Materials and methods

### Immune and metabolism-related genes

Immune-related genes were selected from the IMMPORT database (https://www.immport.org) and InnateDB (https://www.innatedb.com) database. Additionally, metabolism-related genes were independently extracted from the GSEA database. After removing duplicate genes, a total of 2483 immune-related genes and 948 metabolism-related genes were identified. By combining the two sets, 2637 genes were identified as immune- and metabolism-related genes (IMRGs).

### Sample collection and processing

Patient data, including transcriptomic data from 370 tumour specimens and 50 normal specimens, along with detailed clinical and survival data, were obtained from the TCGA (https://portal.gdc.cancer.gov/repository).The exclusion criteria included the histological exclusion of HCC, extremely low gene expression levels, or incomplete clinical data.ID conversion was performed using Perl (Perl programming language version 5.30.1). Liver tissue samples were collected from 20 HCC patients who underwent hepatectomy at Huashan Hospital, Fudan University. All patients were followed up until April 2023 or death. The inclusion criteria for this study were HCC patients without extrahepatic lesions or macrovascular invasion. The exclusion criteria included HCC combined with extrahepatic metastasis or other malignant tumours, death within 3 months after surgery, and incomplete follow-up information. This study was approved by the Institutional Ethics Committee of Huashan Hospital (ethics review number: 2022-903).

### Identification of differentially expressed immune- and metabolism-related genes

To identify differentially expressed genes (DEGs) within immune-related metabolic reprogramming genes (IMRGs) between normal and HCC samples, the “limma” R package (Ritchie et al. 2015) was used for the TCGA-LIHC cohort. The significance criteria were set as |log_2_ FC| > 1 and *P* value <0.05. Subsequently, volcano plots and heatmaps of these DEGs were generated.

### Nonnegative matrix factorization (NMF) consensus cluster for immune- and metabolism-related genes

Based on the expression levels of DEGs, we conducted nonnegative matrix factorization (NMF) clustering analysis using the “NMF” R package (Sharma et al. 2020) (version 0.26.0). The IMRG clusters were identified, and the optimal number of clusters was determined based on NMF clustering results (cophenetic, residual, dispersion, and rss coefficients). Three distinct immune and metabolism clusters were identified in the TCGA-LIHC cohort. The “survival” R package was utilized to assess survival differences among the clusters. A heatmap was generated to visualize the variations between clusters, and the analysis was completed using the “pheatmap” R package (version 1.0.12).

### Assessment of the tumour microenvironment according to molecular subtype

To examine the correlation between the subtypes determined by clustering and the TME, stromal scores, ESTIMATE scores, and immune scores, the “ESTIMATE” package in R was used to derive these scores for all HCC patients. To assess different immune features associated with the clusters, we utilized the “MCPcounter” R package to evaluate the abundance of immune cells. The “limma” R package was used to perform analysis of immune-related genes between clusters, and boxplots and heatmaps were constructed using the “ggpubr” and “pheatmap” packages, respectively.

### Establishment and validation of the immune- and metabolism-related prognostic models

To quantitatively evaluate the intricate interplay between immune and metabolic patterns and HCC, an immune- and metabolism-related prognostic model was constructed. Univariate Cox analysis of the DEGs among the DEGs within the normal and HCC IMRG samples was performed to identify survival-associated genes. Subsequently, we developed a prognostic risk model for immune and metabolism parameters using Lasso-Cox analysis. All TCGA-LIHC HCC samples were divided into a training set (*n* = 259) and a test set (*n* = 111) at a ratio of 7:3. The immune and metabolic risk scores were calculated based on the expression of key genes in the model. The model equation was as follows: Risk score = ∑ni = 1 exp i*coef i (exp i: gene expression, coef: gene risk factor). According to the median risk score, HCC patients were categorized into high- and low-risk groups. KM survival analysis was utilized to examine the differences in survival between the two groups. The accuracy of the model was validated through receiver operating characteristic (ROC) curve analysis.

### Construction and validation of the nomogram for predicting patient prognosis

To investigate the prognostic value of IMRGs, we performed univariate regression analysis and multivariate Cox regression analysis incorporating other clinical and pathological factors, including Tumour grade, age, sex, and TNM stage, to determine their independent risk contribution. Variables with a *P* value <0.05 in the multivariate Cox regression analysis were considered independent risk factors and utilized for constructing a prognostic scoring system using the “rms” R package. The performance of the scoring system was assessed through calibration curve analysis.

### Analysis of the tumour immune microenvironment

Based on the RNA sequencing data from TCGA samples, the CIBERSORT algorithm was utilized to quantify the proportions and abundances of Tumour-infiltrating immune cells (TIICs) (Newman et al. 2015). The LM22 signature algorithm was employed to calculate the abundances of 22 types of TIIC cells. The standardized CRC gene expression profile was transformed into the proportions of TIICs.

### Tumour mutation and therapeutic response prediction

The Tumour mutation burden (TMB) is defined as the total number of mutations per megabase in the tumour tissue and is capable of predicting the response to immune checkpoint blockade (Mariathasan et al. 2018; Chan et al. 2019). The TMB of each sample was calculated using a Perl script (https://www.perl.org/) and differences in TMB between risk groups were analysed using Kaplan–Meier (KM) survival curves. Additionally, we assessed drug sensitivity in treating HCC patients using the “pRRophetic” R package (Geeleher et al. 2014).

### Quantitative real-time polymerase chain reaction (qPCR)

Total RNA was extracted from HCC tissues following the instructions of the FastPure Cell/Tissue Total RNA Isolation Kit V2 (Vazyme, China). The PrimeScript™ RT Master Mix (Takara, Japan) was utilized for reverse transcription of total RNA into cDNA. SYBR Green Master Mix (Vazyme, China) was incorporated during the qPCR. The primer sequences used for detection can be found in Supplementary Table S1.

### Immunohistochemical staining

Immunohistochemical staining was performed on paraffin tissue sections obtained from 20 HCC patients. Prior to the staining procedure, the tissue slides were incubated at 60 °C for 20 min on a hot plate and then immersed in xylene(I) and (II) for a total of 25 min to remove the paraffin. The slides were then hydrated using an alcohol gradient, starting with anhydrous ethanol(I) and (II) for 2 min each, followed by immersion in 95%, 80%, and 70% ethanol(I) and (II) for 2 min each. Subsequently, the slides were washed three times with phosphate-buffered saline (PBS) for 3 min each. To retrieve the antigens, the slides were placed in retrieval solution, boiled at 95 °C for 15–20 min and then allowed to cool naturally for at least 20 min until they reached room temperature. The slides were subsequently washed three times with PBS for 3 min each. To prevent nonspecific binding, excess serum blocking solution was added to the slides, which were then incubated at room temperature for 20 min before removing the excess liquid. The slides were then incubated with the primary antibody overnight at 4 °C. After incubation with the primary antibody, the slides were washed three times with PBS for 3 min each. Subsequently, the slides were incubated at room temperature for 1 h with a secondary antibody conjugated to horseradish peroxidase. The slides were subsequently washed again three times with PBS for 3 min each. The actual staining time was determined using a DAB substrate during microscopic examination of the slides. The reaction was terminated by rinsing the slides with tap water for 10 min. For contrast, the slides were counterstained with haematoxylin for 2 min, followed by differentiation with hydrochloric acid alcohol. The slides were then rinsed with water for 10–15 min. Finally, the slides were dehydrated, cleared, mounted using neutral gum near the tissue, and covered with a coverslip. The tissues were then subjected to microscopic examination.

### Cell culture

The human HCC cell line Huh7 was purchased from the Shanghai Cell Bank of the Chinese Academy of Science (Shanghai, China). The cells were cultured in DMEM (MeilunBio, Dalian, China) supplemented with 10% foetal bovine serum (FBS) and grown at 37 °C in a 5% CO_2_ environment.

### CRISPR-Cas9 gene knockout

Briefly, sgRNA strands for the human UCK2 gene were phosphorylated and annealed prior to cloning into the pLKoCas9-U6-BsmBI-chRNA-EFS-Puro plasmid. The following gRNA strands for human UCK2 were used: the top strand, CACCGGCTGATGTGATCATCCCTAG; and the bottom strand, AAACCTAGGGATGATCACATCAGCC. We used a lentiviral vector to express these gRNAs in Huh7 cell lines that constitutively express Cas9, and the cells were selected with puromycin. Mock cell lines were infected with the lentiviral vector without guide RNA.

### Western blot

Total cellular protein extraction was performed using radioimmunoprecipitation assay (RIPA) buffer (Beyotime, Shanghai, China). The following primary antibodies were incubated at 4 °C for 12 h: UCK2 (1:5000, Proteintech) and GAPDH (1:10,000, Proteintech). The secondary antibody (1:20,000, Proteintech) was incubated at room temperature for 2 h. Immunoreactive bands were visualized using an Enhanced Chemiluminescence Detection Kit (Gen-view Scientific Inc., USA).

### Cell proliferation assay

Cell proliferation was assessed using a Cell Counting Kit-8 (CCK-8) assay kit (Vazyme, China). Huh7 cells (5 × 10^3^) were cultured in triplicate in a 96-well microplate (Corning, Incorporated, New York, USA). Subsequently, 10 μl of CCK-8 reagent (diluted in 100 μl of serum-free medium) was added to each well at 24, 48, 72, and 96 h, followed by a 3-h incubation at 37 °C. The absorbance at 450 nm was measured using a multifunction enzyme-linked analyser (Thermo Fisher Scientific) to calculate cell viability.

### Monocyte-derived macrophage stimulation and coculture with Huh7 cells

Peripheral blood was collected from healthy donors. Peripheral blood mononuclear cells (PBMCs) were isolated using Ficoll-Paque PLUS density gradient centrifugation, followed by purification of CD14^+^ monocytes (Sanin, et al. 2022) using a Human CD14 Positive Selection Kit II (STEMCELL). Monocytes were cultured in RPMI 1640 (MeilunBio) supplemented with 10% foetal calf serum (ExCellBio) and 1000 U/mL penicillin‒streptomycin (MeilunBio) for 7 days (Sieweke and Allen 2013; Brauneck et al. 2022). This allows the differentiation of monocytes into monocyte-derived macrophages (moMacs). To study the capacity of the Huh7 cell line to induce the differentiation of moMacs, we cocultured moMacs with the Huh7 cell line in 96-well plates for 3 days, and the phenotype was analysed by flow cytometry.

### Flow cytometry

The cells were collected, washed with 1 × PBS containing 2% FBS and incubated with a mixture of antibodies (BioLegend) against PerCp/Cy5.5-conjugated anti-human CD80, PE-conjugated anti-human CD45, PE/Cy7-conjugated anti-human CD11b and APC/Cy7-conjugated anti-human CD206 for 30 min in the dark at 4 °C. The cells were analysed using a flow cytometer (Attune, Thermo Fisher Scientific), and the data were analysed using FlowJo software (Tree Star, Inc., Ashland, OR).

### RNA sequencing analysis

The RNA libraries were subsequently sequenced by OE Biotech, Inc., Shanghai, China. Differential expression analysis was performed using DESeq2 (Love et al. 2014). A *Q* value <0.05 and a fold change >2 or <0.5 were set as the thresholds for identifying significant DEGs. Hierarchical cluster analysis of DEGs was performed using R (v 3.2.0) to demonstrate the expression patterns of genes in different groups and samples. Heatmaps of upregulated and downregulated genes were generated using the “Pheatmap” package. Pathway analysis via GSEA was also conducted to determine the pathways in which the genes whose expression was markedly enriched (Subramanian et al. 2005; Mootha et al. 2003).

### Statistical analysis

The log-rank test was used to perform Kaplan‒Meier (KM) analysis and compare the differences in survival between the high- and low-risk HCC groups. A *t* test was used for intergroup difference analysis. Additionally, both univariate and multivariate Cox regression analyses were employed to identify OS-related IMRGs and independent prognostic indicators of OS. All the statistical analyses were conducted using Prism (version 8, https://www.graphpad-prism.cn/) and R statistical software (version 4.1.3; https://www.r-project.org/). A *P* value <0.05 was considered to indicate a statistically significant difference (* *P* < 0.05, ** *P* < 0.01, *** *P* < 0.001).

## Results

### Identification of a classification pattern of HCC based on the differentially expressed genes related to immunity and metabolism

**Fig. 1:**
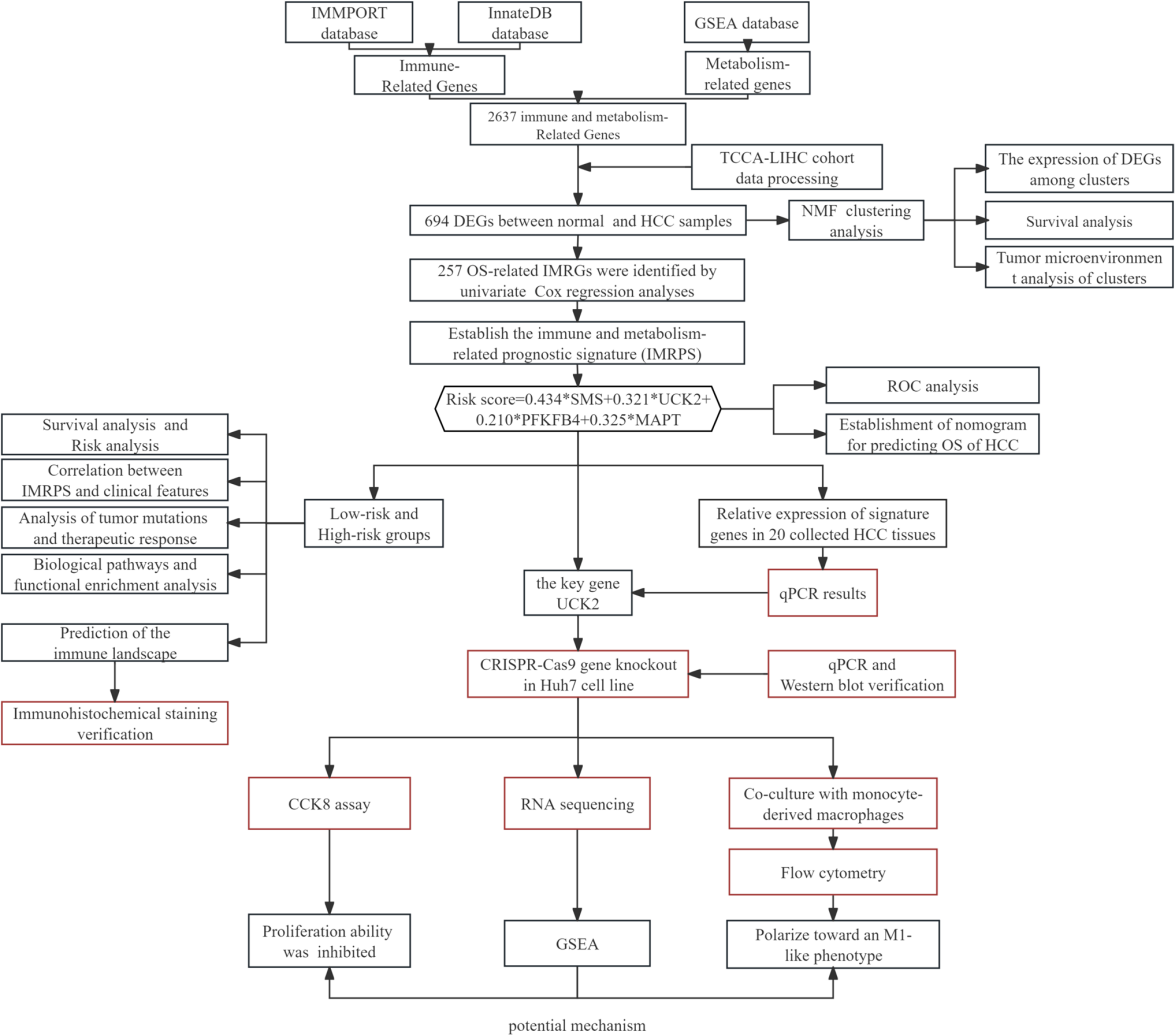
The overall workflow of the current study

**Fig. 2:**
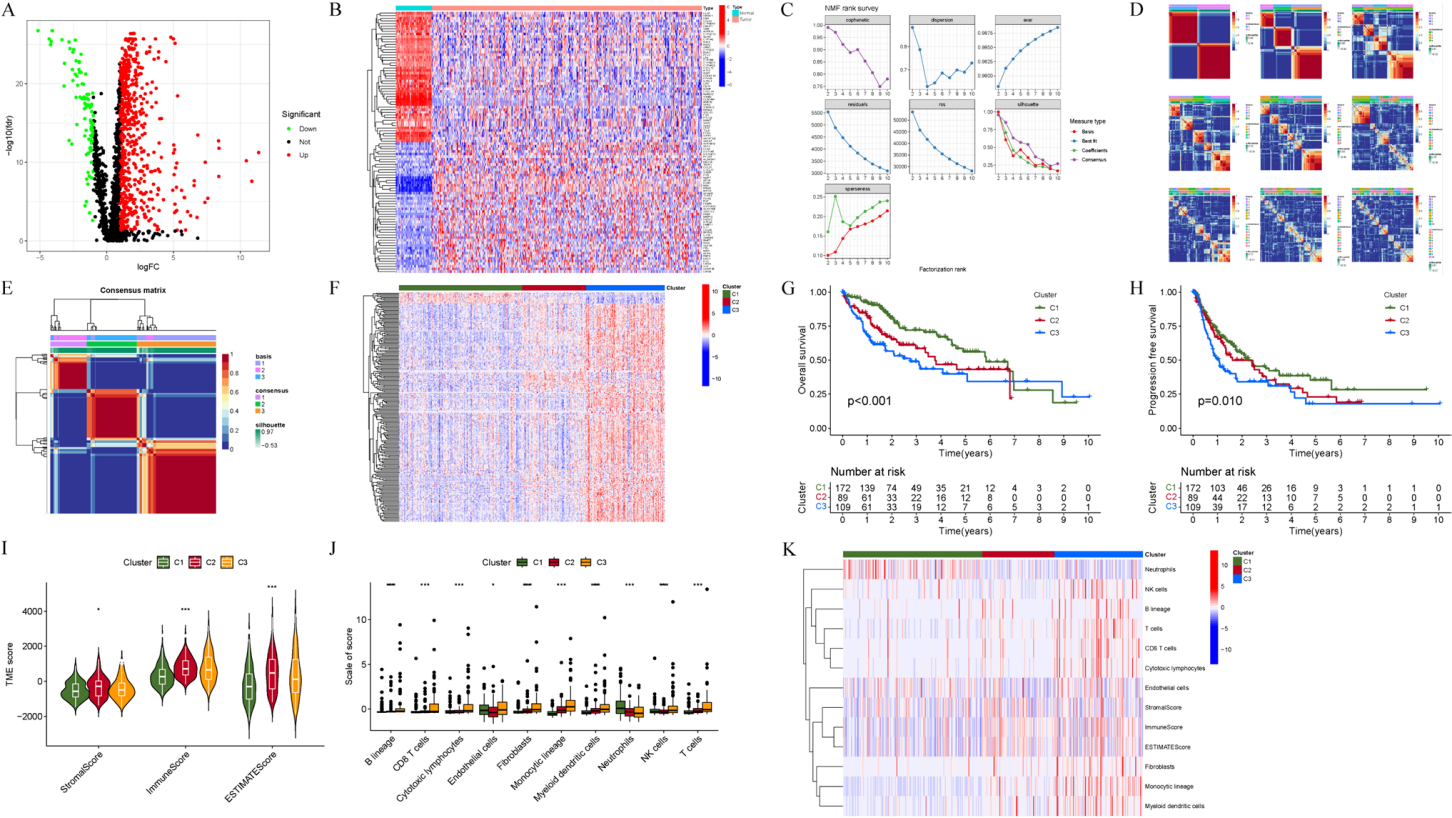
**A**,**B** A total of 694 DEGs within IMRGs between normal samples and HCC samples, with the significance criteria set to |log_2_ FC| > 1 and *P* value <0.05. **C**–**E** Based on the results of NMF clustering (cophenetic, residual, dispersion, evar, silhouette, sparseness and rss coefficients), we selected values of *k* where the magnitude of the cophenetic correlation coefficient began to fall, and three clusters solution best fitted the data (cophenetic correlation >0.9). **F** Heatmap showing the DEGs among the three clusters. **G**,**H** Kaplan‒Meier curves were used to analyse the differences in survival among the clusters. **I** Comparison of stromal scores, estimated scores, and immune scores among the three clusters. **J**,**K** The analysis of differences in the TME among clusters is represented by boxplots and heatmaps

The overall workflow of the current study is displayed in Fig. 1. To explore the expression patterns of IMRGs in HCC, 370 samples from the TCGA-LIHC cohort were clustered using an NMF consensus clustering algorithm based on the expression data of 694 DEGs (Fig. 2A, B). Three clusters, namely, clusters 1 (172 samples), 2 (89 samples) and 3 (109 samples), were obtained by cluster analysis (Fig. 2C–E). Patients in cluster 3 had the worst survival rates according to the Kaplan‒Meier curve (Fig. 2G, H). The heatmap shows the differences among clusters (Fig. 2F).

### TME characteristics and immune infiltration in the three clusters

To clarify the characteristics of the three subtypes of tumours in the TME, immune scores were calculated for the three clusters in this study (Fig. 2I). In contrast to previous findings, the immune score and ESTIMATE score were greater in cluster 3 than in cluster 1. In addition, most immune cells were enriched in cluster 3 (Fig. 2J, K). Among them, CD8^+^ T cells, NK cells and cytotoxic lymphocytes were highly expressed in patients in cluster 3. In addition, macrophages themselves are highly heterogeneous and play dual roles in antitumour immune processes. This may suggest both immune infiltration and immunosuppression in the TME of patients in cluster 3.

### Construction and validation of immune- and metabolism-related prognostic signatures for hepatocellular carcinoma patients

**Fig. 3:**
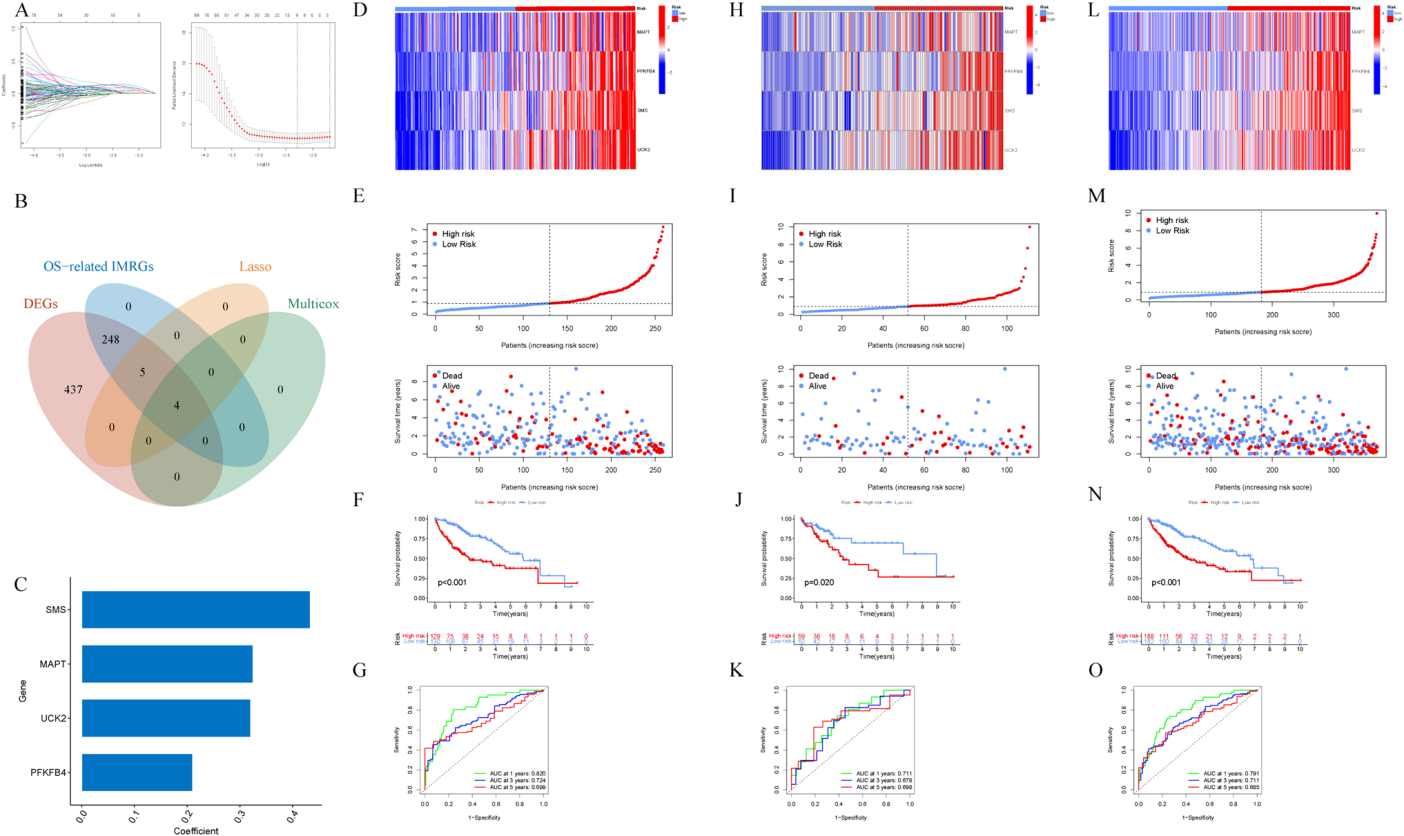
**A**,**B** LASSO Cox regression was performed to identify the optimal IMRGs for establishing the prognostic signature. We obtain the variable coefficients and the optimal number of variables based on the optimal lambda value, and finally obtain the corresponding model with excellent performance and the least number of independent variables. **C** Coefficients of the four signature genes in the immune- and metabolism-related prognostic signatures. **D** Heatmap showing the differences in the expression of signature genes between the low- and high-risk groups. **E** Risk curve analysis showed that increasing risk scores increased the risk of death, and the survival time was relatively short. **F** Kaplan‒Meier survival curves for the low- and high-risk groups. **G** The AUC values of the TCGA training cohort for 1, 3 and 5 years. **H**–**K** The heatmap, risk curve, Kaplan‒Meier survival curve and ROC curve were drawn using the same formula as that for the training cohort. **L**–**O** Heatmap, risk curve, Kaplan‒Meier survival curve and ROC curve for the entire TCGA-LIHC cohort

The analysis of 257 candidate OS-related IMRGs in the TCGA-LIHC cohort was conducted using Lasso Cox regression to identify the optimal IMRGs for establishing an immune- and metabolism-related prognostic signature (IMRPS). Ultimately, four key IMRGs, UCK2, SMS, MAPT, and PFKFB4, which were used to construct the signature, were identified (Fig. 3B, C). Based on these key genes, an immune and metabolic risk score for IMRPS was developed. The risk score = 0.434 × SMS + 0.321 × UCK2 + 0.210 × PFKFB4 + 0.325 × MAPT. By dividing all patients in the TCGA cohort into low-risk and high-risk groups based on the median risk score, it was observed that an increase in the risk score was associated with an increase in the risk of death (Fig. 3D, E). Risk survival analysis revealed that patients in the high-risk group had an increased mortality rate and relatively shorter survival time (Fig. 3E). The KM survival curves for the low-risk and high-risk groups further confirmed these results (Fig. 3F). Additionally, the high accuracy of predicting patient prognosis at 1, 3, and 5 years after surgery was validated for the prognostic signature. The AUC values for the TCGA training cohort were 0.820 at 1 year, 0.724 at 3 years, and 0.699 at 5 years (Fig. 3G). To assess the generalizability of the constructed IMRPS to the validation cohort, risk scores for each patient were calculated using the same formula used for the training cohort. In the validation cohort, patients in the high-risk subgroup exhibited worse survival outcomes than did those in the low-risk subgroup (Fig. 3J). Furthermore, according to the ROC analysis, the AUC values for the IMRPS were 0.711 at 1 year, 0.678 at 3 years, and 0.698 at 5 years (Fig. 3K). Figures 3H and I display the distribution of risk scores, survival status, and expression of the four genes, showing an increase in the mortality rate with increasing risk score.

### Analysis of the correlation between the IMRPS score and clinical features

**Fig. 4:**
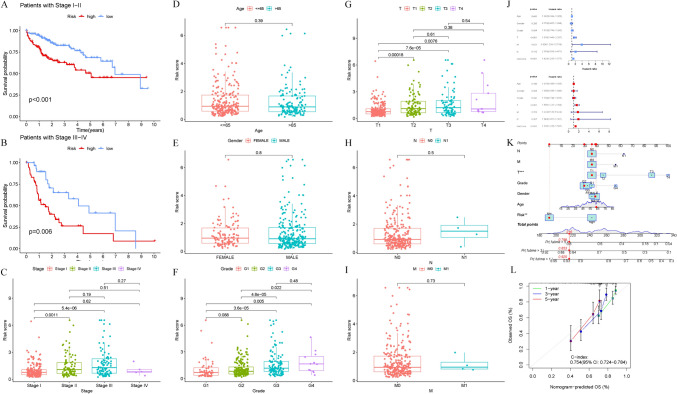
**A**,**B** Patients in the high-risk group were closely linked to poorer outcomes across all stages. **C**–**I** Patients with advanced tumour grade and stage had higher risk scores, but no significant difference was found for age or sex. **J** We conducted univariable and multivariable Cox regression analysis on risk scores and other clinical parameters respectively. Factors with significant *P* values (<0.05) in both univariable and multivariable Cox regression analysis were considered to be independent influencing indicators of OS. Based on the statistical results, we found that the risk score is an independent indicator of OS. **K** The nomogram for OS prediction using clinical parameters and the IMRPS-based risk scores. **L** A calibration plot for validation of the nomogram

We further analysed the value of the IMRPS for two groups of patients with different clinical factors in the TCGA cohort. Higher risk scores were found in patients with advanced tumour grade and stage (Fig. 4C, F–I), but no significant differences were found for age or gender (Fig. 4D, E). Additionally, we also observed a close association between patients in the high-risk group and poorer outcomes across all stages (Fig. 4A, B). Univariate and multivariate Cox regression analyses were conducted to assess whether this signature could serve as an independent predictor of the prognosis of HCC patients. Our findings indicated that the risk score remained an independent indicator of OS (HR = 1.376, 95% CI = 1.225–1.547, *P* < 0.001) after adjusting for other clinical parameters (including age, sex, grade, and TNM stage). Subsequently, we developed a nomogram for predicting OS using clinical parameters and the IMRPS-based risk score in the TCGA cohort (Fig. 4K). A calibration plot demonstrated excellent consistency between the predicted probabilities of 1-year, 3-year, and 5-year OS and the actual observations for the nomogram (Fig. 4L).

### Biological pathway and functional enrichment analysis

**Fig. 5:**
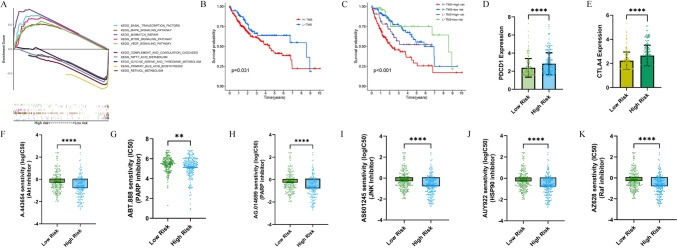
**A** GSEA was performed to explore the most significantly enriched functional pathways between the high-risk and low-risk patients. **B** Survival analysis of patients in the high-TMB subgroup and low-TMB subgroup in the risk model. **C** Survival analysis of patients stratified by the combined TMB and risk score. **D**,**E** Two immunosuppressive checkpoints, CTLA4 and PDCD1, were upregulated in the high-risk group. **F**–**K** Potentially effective inhibitors for high-risk HCC treatment (* *P* < 0.05, ** *P* < 0.01, *** *P* < 0.001, **** *P* < 0.0001)

Given the satisfactory prognostic performance of our IMRPS in HCC patients, we investigated the underlying mechanisms involved. First, differential expression analysis was conducted between the two risk groups (|log_2_-fold change| > = 1, adjusted *P* value <0.05). Moreover, GSEA was performed to further explore the most significantly enriched functional pathways between the high-risk and low-risk patients (Fig. 5A). We found that the activation of oncogenic signalling pathways, such as basal transcription factor, MAPK signalling, mismatch repair, mTOR signalling and VEGF signalling pathways, was increased in the high-risk group. However, complement and coagulation cascades, fatty acid metabolism, glycine serine and threonine metabolism, primary bile acid biosynthesis and retinol metabolism were enriched in the low-risk patients.

### Analysis of tumour mutation burden and therapeutic response prediction

We performed survival analysis on patients in the high-TMB subgroup and low-TMB subgroup within the risk model. The analysis revealed that the OS of patients in the high-TMB and high-risk groups was poorer (Fig. 5B, C). Based on the GSEA results, we investigated potential effective strategies for treating high-risk HCC. We selected several Food and Drug Administration-approved chemotherapeutic agents for analysis. Subsequently, we evaluated the half maximal inhibitory concentrations (IC50) of these inhibitors in the low-risk and high-risk subgroups using the pRRophetic platform. As shown in Fig. 5F–K, high-risk HCC patients exhibited greater sensitivity to PARP, HSP90, AKT, JNK, and Raf inhibitors. These findings suggest that low-risk and high-risk patients may harbour different oncogenic mechanisms that promote the development of HCC. Furthermore, two immune checkpoint inhibitors, CTLA4 and PDCD1, were upregulated in the high-risk group (Fig. 5D, E). Therefore, our immune-mediated risk prediction score (IMRPS) can help in selecting appropriate treatment strategies.

### Prediction and validation of the immune landscape in the IMRPS risk groups

**Fig. 6:**
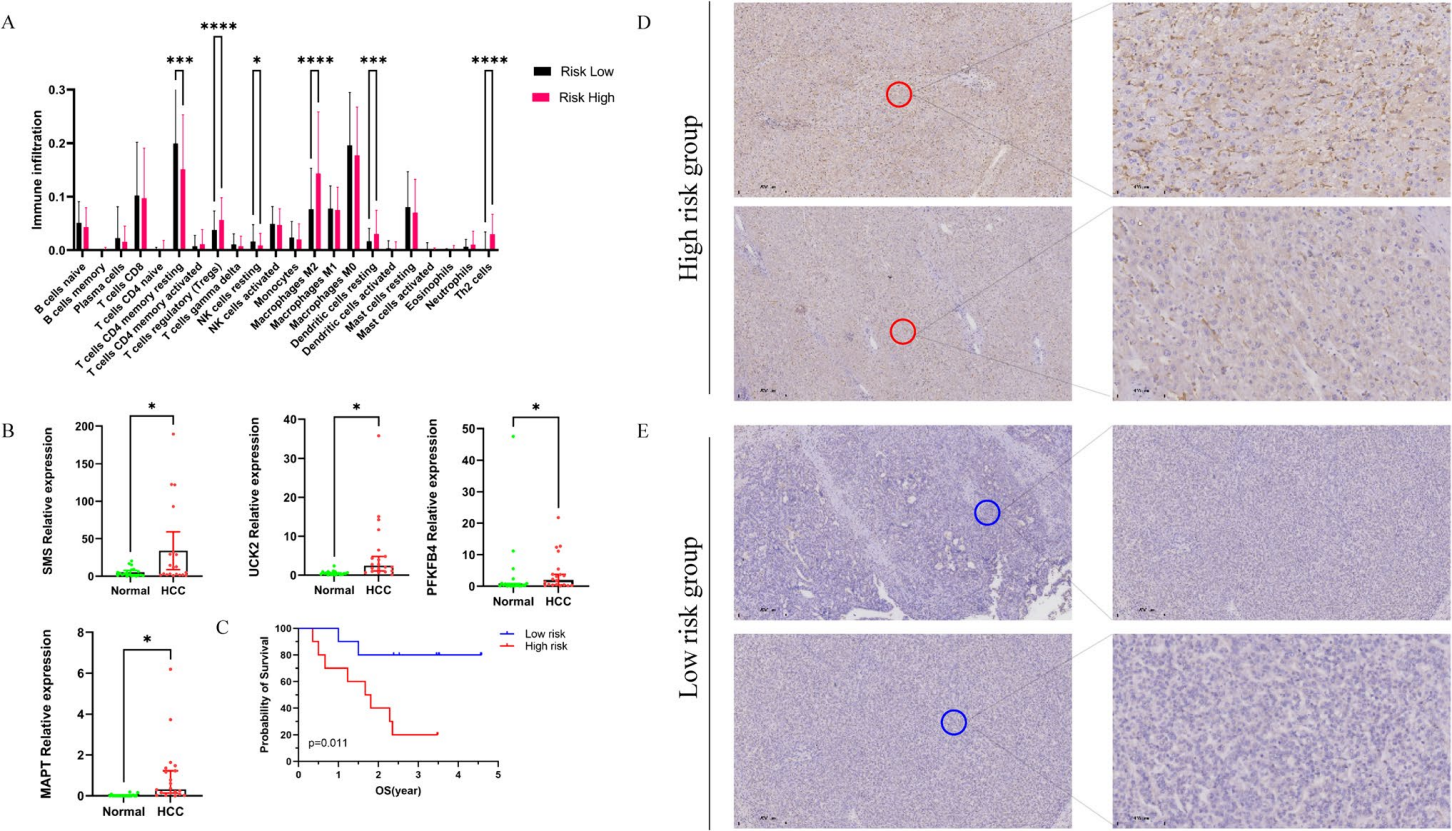
**A** Differences in tumour-infiltrating immune cells (TIICs) between the low- and high-risk groups. **B** The results of qPCR showed that the relative expression of UCK2, SMS, MAPT and PFKFB4 in 20 collected HCC tissues increased compared with that in adjacent normal tissues, and high-risk HCC patients had shorter survival times. **C**,**D** Differences in M2-like macrophage immune infiltration between the low- and high-risk groups

To explore the correlation between the IMRPS and the immune landscape of the HCC samples, the differences in immune cell infiltration between the low- and high-risk groups were compared. CIBERSORT was used to predict the proportion of TIICs in each tissue sample (Fig. 6A). Components with significant differences (*P* < 0.05) were selected for subsequent analysis. M_2_-like macrophages, Tregs, resting dendritic cells and Th_2_ cells were abundant in the high-risk group. In contrast, CD4^+^ resting memory T cells and resting natural killer cells were more predominant in the low-risk group. M_2_-like macrophages, Tregs and Th_2_ cells are important components of the TME. They play essential roles in the regulation of tumour immunity and promote an immunosuppressive microenvironment. Among them, M_2_-like macrophages were most significantly different between the high- and low-risk groups and had the largest relative proportion of infiltrating immune cells. To confirm and validate these findings, immunohistochemical staining was performed to evaluate M2-like macrophage immune infiltration features in HCC tissue samples (Fig. 6D, E). Few M_2_-like macrophages (brown) were observed in the low-risk HCC samples. Consistently, in high-risk HCC samples, there was considerable infiltration of M_2_-like macrophages within the TME. Hence, these results indicate that the IMRPS can characterize the tumour immune microenvironment and that high-risk HCC patients may develop an immunosuppressive microenvironment with abundant M_2_-like macrophage infiltration.

### Verification of the gene expression pattern of the IMRPS in HCC tissues by quantitative real-time PCR

The results of quantitative real-time PCR (qPCR) showed that the relative expression of UCK2, SMS, MAPT and PFKFB4 in 20 HCC tissues was significantly greater than that in adjacent normal tissues (Fig. 6B). The dysregulation of the four genes indicated their potential role as biomarkers in the prognosis and progression of HCC. In addition, the IMRPS risk score was computed for each patient. The 20 patients were classified into low- and high-risk groups according to the median cut-off value of the risk score. The relatively short OS of the high-risk patients was confirmed by Kaplan–Meier (KM) survival curves (Fig. 6C).

### UCK2 knockout inhibited HCC cell proliferation

**Fig. 7:**
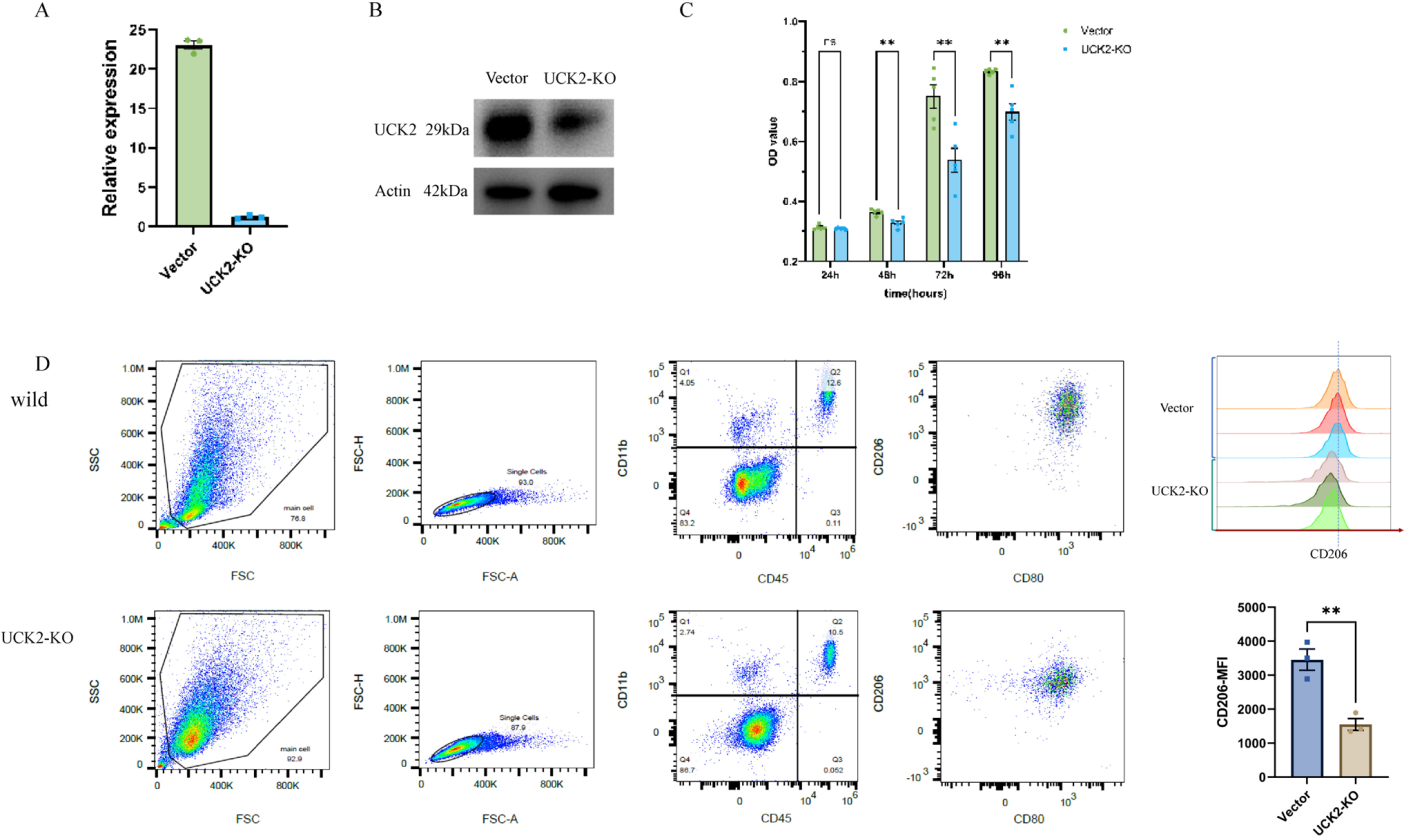
**A**,**B** qPCR and western blot results showing the efficiency of UCK2 knockout. **C** The proliferation of UCK2 knockout cells was significantly inhibited, as evaluated by a CCK-8 assay. **D** UCK2 knockout promoted monocyte-derived macrophage polarization towards an M1-like phenotype, characterized by strong downregulation of CD206. The *P* value of the difference between two groups is 0.0061

UCK2 was knocked out in Huh7 cells by infection with a lentiviral vector to express the gRNAs. The qPCR and western blot results indicated the efficiency of the UCK2 knockout (Fig. 7A, B). Moreover, the proliferation of UCK2 knockout cells was significantly inhibited, as evaluated by a CCK-8 assay (Fig. 7C).

### UCK2 knockout Huh7 cells promoted monocyte-derived macrophage polarization towards an M1-like phenotype

The surface expression of the prototypical M1 marker CD80 and the M2 marker CD206 was subsequently analysed via flow cytometry to confirm polarization. Flow cytometry analysis of monocyte-derived macrophages cocultured with UCK2-KO Huh7 cells revealed the typical M1 phenotype, characterized by strong downregulation of CD206(Fig. 7D). These findings suggest direct modulation of the M1 macrophage phenotype.

### Reflecting the potential mechanism by which UCK2 affects proliferation and monocyte-derived macrophage polarization through RNA-seq

**Fig. 8:**
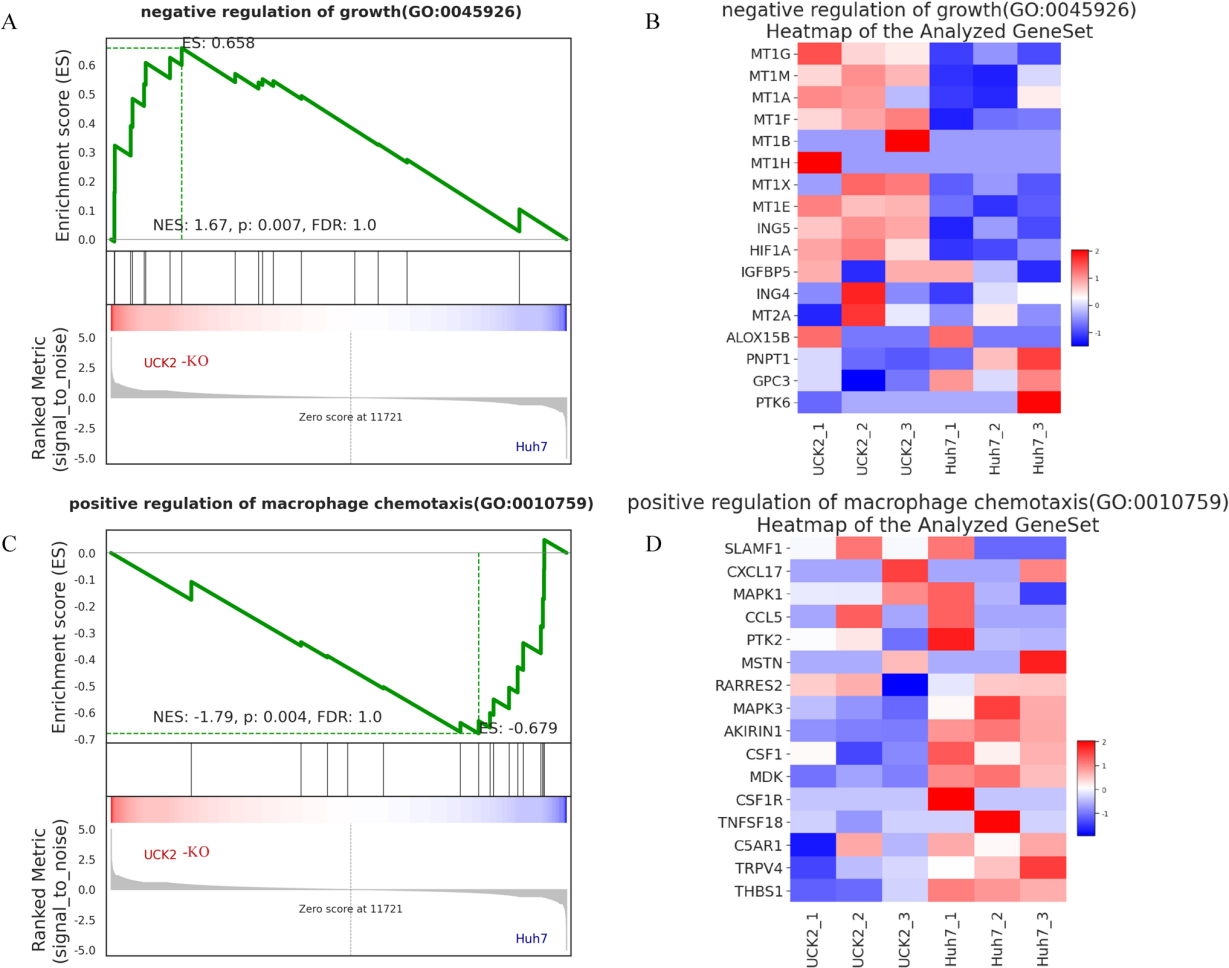
RNA sequencing data and GSEA functional enrichment analysis results. **A** Negative regulation of growth pathways was upregulated in the UCK2-KO group. **B** Heatmap of the negative regulation of growth pathways. **C** The pathway involved in the positive regulation of macrophage chemotaxis was downregulated in the UCK2-KO group. **D** Heatmap of the positive regulation of the macrophage chemotaxis pathway

To further investigate the potential mechanism through which UCK2 affects proliferation and monocyte-derived macrophage polarization, we isolated total RNA from the NT and UCK2-KO Huh7 cell lines. RNA-seq data were analysed, and 189 DEGs were identified. GSEA was subsequently conducted for these DEGs. GSEA suggested that the DEGs were closely related to several pathways related to proliferation and the regulation of macrophage chemotaxis (Fig. 8A–D). The results suggested that the negative regulation of the growth pathway was upregulated and the positive regulation of macrophage chemotaxis pathway was downregulated in the UCK2-KO group, which may explain the inhibited proliferation ability of UCK2 knockout cells and modulation of the M1 macrophage phenotype. Taken together, these findings suggest that UCK2 might play a nonnegligible role in the development of HCC.

## Discussion

Cancer metabolism not only plays a crucial role in cancer signalling to sustain Tumourigenesis and survival (Martínez-Reyes and Chandel 2021; Ge et al. 2022) but also has wider implications for the regulation of the antitumour immune response through both the release of metabolites and the expression of immune molecules (Stine et al. 2022; Pascual et al. 2024).

The use of different features can reveal the clinical relevance and prognostic value of HCC subtypes (Chaisaingmongkol et al. 2017). Our study classified patients into 3 immune- and metabolism-related subtypes based on NMF clustering in an attempt to identify new feature patterns related to tumour progression and treatment in HCC. There were significant prognostic differences among the different subtypes. We believe that immune and metabolic gene signatures are promising biomarkers with tremendous potential. Then, four key genes (UCK2, SMS, PFKFB4 and MAPT) among the survival-related DEGs were screened to establish prognostic features related to immunity and metabolism by analysing the TCGA-LIHC transcriptome and clinical data from HCC samples. We confirmed that high-risk HCC patients had shorter survival times than low-risk patients and were characterized by an immunosuppressive TME. Consistent with these findings, we also observed the same survival curve characteristics in the cohort of 20 patients from Huashan Hospital. Additionally, via immunohistochemical staining, we found abundant M_2_ macrophage infiltration in high-risk samples from real-world experiments. Our results also showed that the risk score of the IMRPS remained an independent indicator of OS after adjusting for other clinical parameters.

There are certain correlations between TMB and tumour prognosis and survival (Cheng et al. 2023). Tumour patients with a high TMB have a better prognosis when receiving immunotherapy (Cheng et al. 2023; Cao et al. 2019; Cao et al. 2022). In our study, high-risk patients had a greater TMB, and the combination of the TMB and risk score could be used to evaluate the prognosis of patients more effectively. Subsequently, we discussed the role of high and low risk in the immunotherapy response. The expression of two immunosuppressive checkpoint proteins, CTLA4 and PDCD1, was upregulated in the high-risk group, which may explain the poor prognosis of these patients (Lei et al. 2020). In general, immune- and metabolism-related prognostic signatures play important roles in independently predicting patient prognosis, tumour immune infiltration and the TMB. According to the GSEA results, the low- and high-risk groups may have different oncogenic mechanisms promoting HCC development. High-risk HCC patients may have elicited better responses to PARP, HSP90, AKT, JNK and Raf inhibitors.

The qPCR results confirmed that UCK2, SMS, PFKFB4 and MAPT were expressed at higher levels in HCC tissues than in adjacent normal tissues (*P* < 0.05). According to the qPCR results, UCK2 (uridine-cytidine kinase 2) was highly expressed in almost all 20 tumour specimens from HCC patients; therefore, we further explored the function and mechanism of this gene. UCK2 is a key regulator of pyrimidine metabolism and catalyses the phosphorylation of uridine and cytidine to form uridine monophosphate and cytidine monophosphate (Zhang and Kleiner 2019), respectively. UCK2 levels are elevated during HCC development, and UCK2 exerts carcinogenic effects. These effects promote cancer cell proliferation and metastasis by activating the EGFR–AKT signalling pathway (Cai et al. 2020). Consistent with the findings of previous studies, we found that the proliferation of Huh7 cells was significantly inhibited when UCK2 was knocked out (Wu et al. 2022). However, the link between UCK2 and the TME has not been investigated. The M_2_-like macrophage population exhibited the most significant difference between the high- and low-risk groups in the TME and had the largest relative proportion of infiltrating immune cells. Therefore, we speculate that the high expression of UCK2 in HCC tissues is an important driving factor for the infiltration of M2 macrophages in the TME. Flow cytometry analysis of monocyte-derived macrophages cocultured with UCK2-KO Huh7 cells revealed that the M1 macrophage phenotype was modulated. Therefore, UCK2 may be a biomarker of the immunosuppressive microenvironment. Moreover, the downregulation of UCK2 also improved the immunosuppressive microenvironment.

To further investigate the potential mechanism through which UCK2 affects proliferation and monocyte-derived macrophage polarization, RNA sequencing data were analysed. The GSEA results suggested that the expression of genes associated with the negative regulation of growth pathways was upregulated in the UCK2-KO group, which may explain the inhibited proliferation ability of these cells. However, the pathways associated with the positive regulation of macrophage chemotaxis, especially the two key genes CSF1 and MDK, were downregulated in the UCK2-KO group. According to previous research, HCC-derived CSF1 polarizes macrophages toward the M2 phenotype to drive immune escape and anti-PD1 tolerance (Wei et al. 2022). An increase in MDK results in an interaction with its receptor LRP1, which is expressed by tumour-infiltrating macrophages and promotes immunosuppressive macrophage polarization (Zhang et al. 2021). To some extent, these findings may explain the high expression of UCK2, which promotes the infiltration of many M2 macrophages in the TME.

However, our study has several limitations. Firstly, additional independent cohorts and a larger sample size are needed to validate the accuracy of the model. Secondly, our study is retrospective in nature, focusing on HCC, and further basic experiments are necessary to verify the underlying mechanisms, which will be the direction of our future endeavors.

In summary, we established prognostic features related to immune and metabolic parameters for stratifying high-risk HCC patients. Additionally, the developed biomarkers can serve as novel indicators for determining patient prognosis and treatment. Furthermore, the study identified key prognostic genes associated with the progression of HCC and validated their expression. The role of the key gene UCK2 in HCC progression and the immunosuppressive microenvironment has been elucidated through immunohistochemistry, gene knockout, CCK8, and flow cytometry assays, and to some extent, the specific mechanism has been revealed through RNA-seq analysis. Overall, this research provided a new immune- and metabolism-related model to develop accurate prognosis prediction, which is also advantageous in identifying new directions for HCC treatment.

### Supplementary Information

Below is the link to the electronic supplementary material.

Supplementary file1 (DOCX 15 KB)

## Data Availability

This study utilized publicly available datasets for analysis. The website addresses are as follows: https://www.immport.org; https://www.innatedb.com; https://portal.gdc.cancer.gov/repository. Data from Huashan Hospital can be provided upon reasonable request.
