# Supplementary material for: Immune- and metabolism-related gene signature analysis uncovers the prognostic and immune microenvironments of hepatocellular carcinoma
Source: J Cancer Res Clin Oncol. 2024 Jun 19;150(6):311. doi: 10.1007/s00432-024-05849-5 (PMC11186947; doi:10.1007/s00432-024-05849-5)
Supplement: Supplementary file 1 — Supplementary file1 (DOCX 15 KB) [file 432_2024_5849_MOESM1_ESM.docx]

**Supplementary Table S1** The sequence of primers used in Quantitative real-time PCR (qPCR)

| **Genes** | Forward primers | Reverse primers |
| --- | --- | --- |
| SMS | 5’-AGAGTTATGATGGTGATGCGCAA-3’ | 5’-TCCTCGCACTATGGGTGGTAA-3’ |
| UCK2 | 5’-AGAATGAGGTGGACTATCGCCA-3’ | 5’-GTTCATTGTCAAAGGCATCCGG-3’ |
| MAPT | 5’-AAACCTCTGATGCTAAGAGCACT-3’ | 5’-GTCTCCAATGCCTGCTTCTTCA-3’ |
| PFKFB4 | 5’-TACCTGAACTGGATTGGTGTGC-3’ | 5’-TCCTGATTTTCAGGCCCTCTTC-3’ |
| GAPDH | 5’-GTCTCCTCTGACTTCAACAGCG-3’ | 5’-ACCACCCTGTTGCTGTAGCCAA-3’ |
